# Acetylcholine Receptor Activation as a Modulator of Glioblastoma Invasion

**DOI:** 10.3390/cells8101203

**Published:** 2019-10-05

**Authors:** Emily G. Thompson, Harald Sontheimer

**Affiliations:** 1 Glial Biology in Health, Disease and Cancer Center, Fralin Biomedical Institute at Virginia Tech-Carilion, Roanoke, VA 24016, USA; egthomp@vt.edu; 2 Department of Neurobiology, University of Alabama at Birmingham, Birmingham, AL 35294, USA; 3 School of Neuroscience, Virginia Polytechnic Institute and State University, Blacksburg, VA 24061, USA

**Keywords:** glioblastoma, invasion, acetylcholine receptors, matrix metalloproteinases

## Abstract

Grade IV astrocytomas, or glioblastomas (GBMs), are the most common malignant primary brain tumor in adults. The median GBM patient survival of 12–15 months has remained stagnant, in spite of treatment strategies, making GBMs a tremendous challenge clinically. This is at least in part due to the complex interaction of GBM cells with the brain microenvironment and their tendency to aggressively infiltrate normal brain tissue. GBMs frequently invade supratentorial brain regions that are richly innervated by neurotransmitter projections, most notably acetylcholine (ACh). Here, we asked whether ACh signaling influences the biology of GBMs. We examined the expression and function of known ACh receptors (AChRs) in large GBM datasets, as well as, human GBM cell lines and patient-derived xenograft lines. Using RNA-Seq data from the “The Cancer Genome Atlas” (TCGA), we confirmed the expression of AChRs and demonstrated the functionality of these receptors in GBM cells with time-lapse calcium imaging. AChR activation did not alter cell proliferation or migration, however, it significantly increased cell invasion through complex extracellular matrices. This was due to the enhanced activity of matrix metalloproteinase-9 (MMP-9) from GBM cells, which we found to be dependent on an intracellular calcium-dependent mechanism. Consistent with these findings, AChRs were significantly upregulated in regions of GBM infiltration in situ (Ivy Glioblastoma Atlas Project) and elevated expression of muscarinic AChR M_3_ correlated with reduced patient survival (TCGA). Data from the Repository for Molecular Brain Neoplasia Data (REMBRANDT) dataset also showed the co-expression of choline transporters, choline acetyltransferase, and vesicular acetylcholine transporters, suggesting that GBMs express all the proteins required for ACh synthesis and release. These findings identify ACh as a modulator of GBM behavior and posit that GBMs may utilize ACh as an autocrine signaling molecule.

## 1. Introduction

Glioblastomas (GBMs) are the most common primary malignant brain tumors in humans, occurring in approximately 13,000 people annually in the United States [1]. Long-term patient survival is rare, with around 85 percent of patients succumbing to the disease within two years [1]. Moreover, the current median patient survival rate is only 12 to 15 months [1,2]. This poor prognosis occurs despite aggressive surgical resection of the primary tumor mass, numerous rounds of radiation, and treatment with the chemotherapeutic agent temozolomide [3]. Ultimately, it is the rampant infiltration of GBM cells into the unaffected brain tissue surrounding the tumor mass that is largely responsible for tumor reoccurrence and the limited efficacy of current standard-of-care treatment. Thus, it is imperative that our approach to improving overall survival from these deadly tumors is rooted in understanding the signaling axes that govern this GBM behavior.

The cellular origin of GBMs has long been a point of contention, with debate centering on whether these tumors are the result of the dedifferentiation of mature cells or the accumulation of oncologic mutations in neural stem cells (NSCs) [4]. Several recent studies have strongly suggested that GBMs arise from NSCs that are still present in the adult brain [5,6]. NSCs are found in limited quantities in restricted sites in the adult brain, namely the subventricular zone (SVZ) of the lateral ventricle and the subgranular zone (SGZ) of the hippocampal dentate gyrus [7]. Efforts have been made to understand the factors that regulate and maintain the NSC population in the adult brain, where neurotransmitters and their receptors have been strongly implicated [7,8,9,10]. Whether GBM-initiating cells retain their responsiveness to neurotransmitters at their genesis and throughout tumor progression, however, remains poorly understood.

Interestingly close to 90% of glioblastomas develop in the cerebral lobes, a microenvironment that differs significantly from the microenvironment of the proposed site of initiation in the SVZ or SGZ [5,11]. The cerebral cortex is richly innervated by neurotransmitter projections originating from deep structures in the brain such as the thalamus, amygdala, ventral tegmental area, hippocampus, and basal forebrain. The migration of NSCs has been shown to be modulated by neurotransmitters [12]. As such, if GBM cells have maintained their responsiveness to neurotransmitters this could play a role in the biology of these tumors within the microenvironment of the cortex where they establish and aggressively infiltrate. Indeed, recent studies suggest that GBM cells are able to sense monoamine neurotransmitters, serotonin, and dopamine, with implications on the proliferative ability of GBMs [13,14]. Furthermore, a role for glutamate has been well characterized [15]. GBM cells are not only responsive to glutamate, but also use the system x_c_^−^ cystine–glutamate transporter to elevate peritumoral glutamate levels to excitotoxic levels for surrounding neurons [16,17,18,19]. The role of the classic neurotransmitter acetylcholine (ACh) and its receptors (AChRs) in GBM, on the other hand, has had limited and conflicting investigation [20,21]. Determining if these tumors are responsive to ACh, which can be found in the areas that these tumors preferentially grow, could provide important, novel insights into the interplay of neurotransmitters and GBM biology. Here, we present a comprehensive study investigating the expression and function of AChRs in GBM.

## 2. Materials and Methods

### 2.1. Analysis of GBM Cohort Datasets

**Figure 1:**
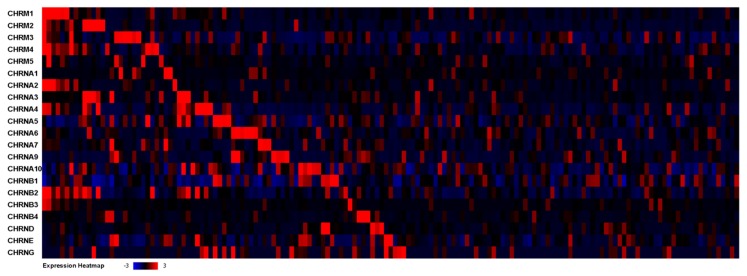
AChR expression in the TCGA dataset. RNA-Seq mRNA expression heatmap of AChR genes across GBM patients in the TCGA cohort (*n* = 156 GBM samples) via the cBioPortal platform.

The publicly available GBM datasets (TCGA [22], REMBRANDT [23], Ivy GAP [24]) were accessed, analyzed, and plots were created via the GlioVis portal (gliovis.bioinfo.cnio.es [25]) with the exception of Figure 1, where cBioPortal [26] was utilized.

### 2.2. Cell Lines

D54 and U251 glioblastoma cells (WHO Grade IV) were gifts from Dr. D. Bigner (Duke University, Durham, NC, USA), and Dr. G. Yancey Gillespie (University of Alabama at Birmingham, Birmingham, AL, USA), respectively. Previously generated stable eGFP-expressing daughter lines (D54-eGFP and U251-eGFP) were used in several experiments. Cells were grown in Dulbecco’s modified Eagle’s medium/Nutrient Mixture F-12 medium (DMEM/F-12) supplemented with 2 mM l-glutamine (ThermoFisher Scientific, Waltham, MA, USA) with 7–10% fetal bovine serum (FBS; Aleken Biologicals, Texarkana, TX, USA) at 37 °C and 10% CO_2_.

### 2.3. Patient-Derived Xenograft (PDX) Tumor Lines

The PDX14 and PDX22 lines were obtained from Dr. Yancey Gillespie (Brain Tumor Tissue Core, University of Alabama at Birmingham, Birmingham, AL, USA). All PDX lines were maintained by serial passage in the flank of athymic nude mice, as previously described [27]. In short, tumor tissue was harvested after 2–3 weeks of flank propagation. The tissue was homogenized into small pieces and resuspended in phosphate-buffered saline (PBS) and ~200 μL of the solution was injected subcutaneously into the flank of an athymic nude mouse for tumor propagation. The remaining tissue was dissociated with a GentleMACS Tumor Dissociation Kit (MACS Miltenyl Biotec, Bergisch Gladbach, Germany) and maintained as a suspension culture in DMEM/F12 medium supplemented with 10 ng/mL EGF and FGF, 250 μg/mL amphotericin, 50 mg/mL gentamycin, 2% B-27 supplement without vitamin A and 1 mM sodium pyruvate (ThermoFisher Scientific). Xenograft cells were used for various experiments after 4–10 days in culture.

### 2.4. RNA Isolation, RT-PCR, and Quantitative PCR

Total RNA was isolated from GBM cell lines (D54 and U251), two patient-derived xenograft lines (PDX14 and PDX22), and control brain tissue from human cortex using the Purelink RNA Mini Kit (ThermoFisher Scientific). cDNA was then synthesized using SuperScript VILO Master Mix (ThermoFisher Scientific). The expression of AChRs was determined using quantitative real-time PCR (qPCR). Each cDNA sample was amplified using Taqman reagents (ThermoFisher Scientific) on an ABI StepOnePlus Real-time PCR System. Taqman probes were as follows: CHRM1 Hs00265195_s1, CHRM2 Hs00265208_s1, CHRM3 Hx00265216_s1, CHRM4 Hs00265219_s1, CHRM5 Hs00255278_s1, CHRNA3 Hs01088199_m1, CHRNA4 Hs00181247_m1, CHRNA5 Hs00181248_m1, CHRNA7 Hs01063372_m1, CHRNB2 Hs00181267_m1, CHRNB3 Hs00181269_m1, CHRNB4 Hs00609520_m1, and IPO8 Hs00914057_m1. Expression in all cell and PDX lines was confirmed by three independent samples, and all samples were analyzed in triplicate.

### 2.5. Time-Lapse Calcium Imaging

D54 and U251 cells were plated on coverslips in DMEM/F12 medium. The PDX lines were plated on coverslips pre-coated with poly-ornithine (Sigma-Aldrich). The coverslips were then loaded with a cell-permeant Fluo-4AM (ThermoFisher Scientific) solution in aCSF (125 mM NaCl, 3 mM KCl, 25 mM NaHCO_3_, 25 mM glucose, 1.25 mM NaH_2_PO_4_, 2 mM MgCl_2_, and 2 mM CaCl_2_) (Sigma-Aldrich, St. Louis, MO, USA) for 10 min in an incubation chamber. The dye solution was then removed and the cells were allowed to recover for at least 30 min before imaging began. The coverslips were then moved into a perfusion chamber that allowed for temperature, aCSF flow-rate, and gas control on an Olympus F1000V laser scanning microscope. After 10 min of equilibration time in aCSF, the GBM cells were exposed either to aCSF only (control) or AChR agonists or antagonists following at least two minutes of baseline in aCSF only. Ca^2+^ dynamics in response to drug application were recorded by time-lapse imaging (1 image/ 3 s). Data analysis was performed using open-source ImageJ Fiji software [28] where a region of interest (ROI) was defined per cell and changes in fluorescent intensity (ΔF/F) were calculated relative to the aCSF baseline (2 min), prior to introduction of the AChR modulators. The response of at least 10 cells was measured and graphed per condition.

### 2.6. Proliferation Assay

Cells were plated at 100,000 per well in a 24-well plate and alamarBlue reagent (ThermoFisher Scientific) was added per the manufacturer’s instructions. The plates were read on a spectrophotometer (Biotek Epoch 2, Winooski, VT, USA) at the specified timepoints. The percentage of reduced reagent was calculated and used as a read out of cell proliferation, per the manufacturer’s instructions. All experiments were performed in triplicate.

### 2.7. Transwell Migration Assay

BD Falcon Fluoroblok transwell filters (ThermoFisher Scientific) with 8-μm pores were placed into 24-well plates. Cells used in the assay were serum-starved (U251) or growth factor deprived (PDX14) for at least 24 h before the start of the experiment. In the well below the filter, DMEM/F12 supplemented with 10% FBS and drugs at specified concentrations were added while 100,000 cells were resuspended in DMEM/F12 with the addition of drugs at specified concentrations and placed on the top of the filter. Cells were then allowed to migrate for 12–48 h. At the specified timepoint, the filters were washed with PBS and fixed with 4% PFA overnight at 4 ℃. A DAPI stain was used to identify cells that had fully migrated to the bottom side of the filter. Five fields were imaged on the bottom of each filter with a Fluoview 1000 confocal microscope using a 20× 0.75NA objective. The number of migrated cells was counted using ImageJ Fiji software. All experiments were performed in triplicate at minimum.

### 2.8. Immunocytochemistry

The D54 and U251 GBM cell lines were cultured on glass coverslips overnight in DMEM/F12 medium supplemented with FBS. Single cells from dissociated xenograft cell cultures were plated on glass coverslips pre-coated with poly-l-ornithine. The coverslips were fixed with a 4% PFA solution for 10 min at room temperature, washed with cold PBS 3 times for 5 min, and permeabilized (PBS with 0.25% Triton X-100) for 10 min. The coverslips were blocked (PBS with 10% donkey serum and 0.1% Tween-20) for 30 min. Primary antibody mouse anti-MMP9 (Abcam, ab119906, Cambridge, United Kingdom) and sheep anti-ChAT (Abcam ab18736) were diluted 1:100 in blocking solution and applied at room temperature for 1 h. The coverslips were washed 5 times for 5 min with PBST (PBS with 0.1% Tween-20). Secondary antibody Donkey anti-Mouse Alexa Fluor 555 (ThermoFisher Scientific) and DAPI diluted in blocking solution were applied at room temperature for 1 h. The coverslips were washed 5 times for 5 min with PBST and then mounted with Fluoromount G Mounting Solution (Electron Microscopy Sciences Cat. #17984-25, Hatfield, PA, USA). Images were acquired using an A1R Nikon laser scanning microscope with a Plan Apo 60×/N.A.1.40 oil objective.

### 2.9. MMP Quantification-Sample Preparation

The D54 and U251 cell lines were plated in 100 mm dishes containing DMEM/F12 supplemented with 7% FBS. At 70–80% confluency, the serum containing medium was removed, the cells were washed twice with PBS, and then DMEM/F12 medium without serum but containing the specified drug concentrations was added. At specified timepoints, the medium was collected and concentrated with a Corning Spin-X UF 6 10k MWCO concentrator (Sigma-Aldrich) at 3400× g for 20 min at 4 °C, while the cells were trypsinized, pelleted, and snap frozen for further analysis. For the PDX lines, the cells were dissociated into a single-cell suspension with Accutase (Sigma-Aldrich), counted, and plated in 6-well plates at 1 × 10^6^ and DMEM/F12 without supplementation, but containing the specified drug concentrations. At the specified timepoint, the medium was collected, stored, and the cells were washed, pelleted, and snap frozen for further analysis.

### 2.10. MMP Quantification-Gelatinase Zymography

Gelatin zymography was utilized for its high sensitivity to detect and quantify gelatinolytic MMP activity. Samples are run in non-reducing conditions to allow for renaturation of the enzymes, and then the subsequent digestion of a gelatin embedded gel can be visualized as clear bands against an intensely stained background. Gelatinase zymography was performed with 10% Novex pre-cast SDS polyacrylamide gel impregnated with 0.1% gelatin (ThermoFisher Scientific). Concentrated conditioned medium or cell lysates were added to the Tris-Glycine SDS sample buffer and loaded for SDS-PAGE with Tris-Glycine SDS buffer. Following electrophoresis, the gel was washed twice in washing buffer (2.5% Triton X-100, 50 mM Tris-HCl, pH 7.5, 5 mM CaCl_2_, and 1 µM ZnCl_2_) for 30 min at room temperature to remove the SDS. The gel was then incubated at 37 °C for 24 h in incubation buffer (1% Triton X-100, 50 mM Tris-HCl, pH 7.5, 5 mM CaCl_2_, and 1 µM ZnCl_2_), and then stained with Coomassie Blue Staining Solution (0.5% Coomassie Blue, 40% methanol, 10% acetic acid, 50% H_2_O) for 45 min. Following destaining (40% methanol, 10% acetic acid, 50% H_2_O), the gel was imaged on an Azure Imaging Station and densitometry measurements were made with ImageJ Fiji software. All experiments were performed in triplicate at minimum.

### 2.11. Transwell Invasion Assay

BD Falcon Fluoroblok transwell filters with 8-μm pores were placed into 24-well plates. The filters were coated with 200 µg/mL Matrigel (Corning, Corning, NY, USA) as directed by the manufacturer for 2 h at 37 °C, followed by a wash with DMEM/F12 medium. Cells used in the assay were serum-starved (U251) or growth factor deprived (PDX14) for at least 24 h before the start of the experiment. In the well below the filter, invasion assay buffer (DMEM/F12 medium supplemented with 0.1% fatty-acid free bovine serum albumin) was added while 100,000 cells were resuspended in DMEM/F12 supplemented with drugs at the specified concentrations and placed on top of the filter. Cells were then allowed to invade for 24–48 h. At the specified timepoint, the filters were washed with PBS and fixed with 4% PFA overnight at 4 ℃. A DAPI stain was used to identify cells that had fully invaded to the bottom of the filter. Five fields were imaged on the bottom of each filter with a Fluoview 1000 Confocal Microscope using a Plan Apo 20× 0.75NA objective. The number of invaded cells was counted using ImageJ Fiji software. All experiments were performed in quadruplicate.

## 3. Results

The focus of this study was to determine if GBMs are responsive to the neurotransmitter ACh and if so, how GBM biology is modulated. As the majority of GBMs grow in areas of the brain that are characterized by the release of neurotransmitters, we hypothesized that GBM cells have the ability to respond to these molecules, specifically ACh, and that this could affect the behavior of GBMs.

### 3.1. Acetylcholine Receptors Are Expressed in Glioblastomas

To examine the expression of AChRs across a large cohort of GBM patients, we utilized The Cancer Genome Atlas (TCGA, https://portal.gdc.cancer.gov/ [22]) dataset of GBM samples. The TCGA dataset affords multi-dimensional analysis of not only the altered genomic landscape of GBMs, but also information about the entire GBM transcriptome through microarray and RNA-Seq analysis. We first queried the dataset to determine the frequency of mutations in the muscarinic AChR (mAChR) genes: *CHRM1-5*; neuronal nicotinic AChR (nAChR) genes: *CHRNA2-7*, *CHRNA9-10*, and *CHRNB1-4*; and muscle nAChR genes: *CHRNA1*, *CHRNB1*, *CHRND*, *CHRNE*, and *CHRNG*. Genomic mutations in these genes were found to be rare, occurring in ≤1% of all GBM samples (Table S1). Next, we wanted to determine the level of AChR expression at the transcriptional level within the TCGA GBM cohort. Utilizing RNA-Seq data from 156 GBM patients, we analyzed the mRNA expression of the AChRs (Table 1, Figure 1). The expression of the majority of the AChRs was found to be at lower levels in comparison to non-tumor samples—as these control samples presumably include a large population of neurons, which have elevated expression of AChRs. However, we did find the expression of *CHRNA1*, *CHRNA9*, and *CHRNB1* to be significantly upregulated in the GBM samples in comparison to the non-tumor samples.

These findings suggest that the AChRs genes are rarely mutated in GBMs. Thus, this allows for transcriptional expression of the AChRs in GBMs, as illustrated in Table 1 and Figure 1, and the potential for GBMs to be responsive to ACh present in the environment in which these tumors grow. Additionally, we used quantitative RT-PCR (qPCR) to characterize the expression of AChRs in two widely available GBM cell lines (D54 and U251), as well as, two patient-derived xenograft lines (PDX14 and PDX22). We confirmed the expression of most neuronal AChRs in these GBM cell and xenograft lines, and utilized them for subsequent experiments in this study (Figure S1).

### 3.2. GBM Acetylcholine Receptors Are Functional

To assess whether the mRNA expression observed with RNA-Seq gene analysis of the TCGA dataset correlated to the expression of functional AChRs, we used an increase in intracellular calcium concentration ([Ca^2+^]_i_) as a readout of AChR activity. Increases in [Ca^2+^]_i_ occur upon activation of both the mAChRs and nAChRs via different signaling cascades [29,30]. mAChRs are G-protein coupled receptors (GPCRs), the M_1_, M_3_, M_5_-subtypes activate G_q_ signaling, leading to the activation of Protein Kinase C (PKC). Ultimately, through the generation of Inositol 1,4,5-triphosphate (IP_3_), Ca^2+^ is released from the endoplasmic reticulum (ER) via IP_3_ Receptor (IP_3_R) activation. nAChRs, on the other hand, are non-selective cation channels that allow the flow of cations, including Ca^2+^ ions, into or out of a cell depending on the ionic driving force. All nAChRs are permeable to Ca^2+^ ions, however, there is ionic conductance variability between nAChRs based on subunit composition [31]. Notably, α7-nAChRs are the most permeant to Ca^2+^ ions [32].

**Figure 2:**
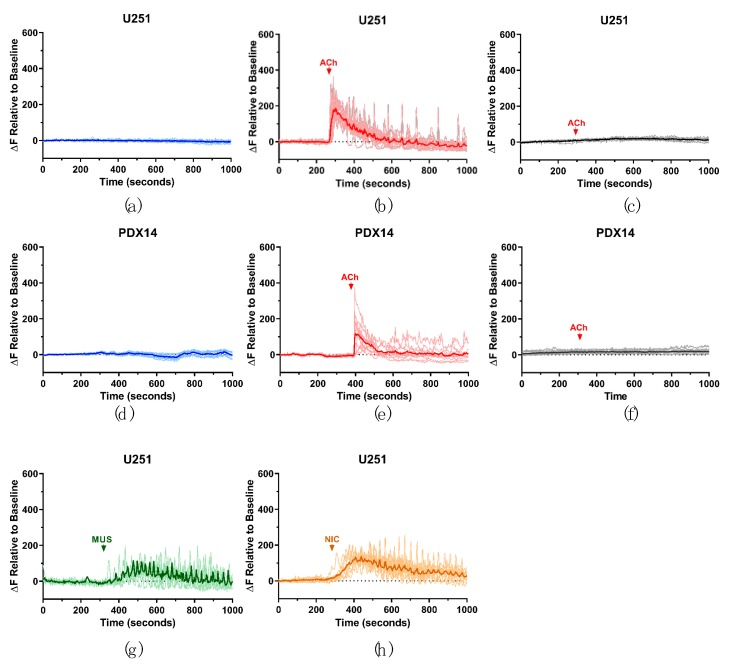
AChR activation causes an intracellular Ca^2+^ increase in GBM cells. Representative graphs of [Ca^2+^]_i_ changes in the U251 and PDX14 lines. GBM cells were loaded with Fluo-4AM. aCSF was perfused into the imaging chamber for at least 10 min to establish a basal Ca^2+^ activity recording (**a**,**d**). Following this baseline period, 1 mM ACh was perfused into the chamber and the response was recorded (**b**,**e**). AChRs were inhibited by the addition of 1 μM atropine and 10 μM mecamylamine for 10 min, then 1 mM ACh was also added to the solution (arrow indicates the presence of ACh in the imaging chamber) and the response was recorded (**c**,**f**). The response of U251 cells to 10 μM muscarine (MUS) (**g**) and 100 μM nicotine (NIC) (**h**). ΔF relative to the baseline was calculated by taking the average fluorescent intensity during the first 2 min of the experiment, prior to drug addition, and making all fluorescent intensities relative to this calculated value. For each experiment, the response of at least 10 cells was quantified, with each individual cell’s response graphed in the lighter line and the averaged cell response shown in the darker line. All experiments were performed at least three separate times as confirmation of the responses shown in the representative graphs.

We utilized confocal time-lapse Ca^2+^-imaging to investigate changes in [Ca^2+^]_i_ following AChR modulation in the GBM cell and xenograft lines (Figure 2). Fluo-4AM loaded cells were perfused with artificial cerebrospinal fluid (aCSF) and the basal Ca^2+^ activity (fluorescence) of the GBM cells was established. All cell lines used in these experiments were responsive to the application of ACh (1 mM), selective mAChR agonist muscarine, and selective nAChR agonist nicotine (U251/PDX14 Figure 2; D54/PDX22 Figure S2). This response was characterized by a large initial spike in [Ca^2+^]-dependent fluorescence, followed in some cases by smaller oscillations (Figure 2b,e). To demonstrate the specificity of this response to AChR activation alone, AChR inhibitors (AChRIs, atropine and mecamylamine) were used in combination with AChR agonists. For example, AChRIs were perfused onto the cells for 10 min, after which the response to ACh was recorded. Under these conditions, no change in Ca^2+^ intensity was observed (Figure 2c,f). Additionally, to ensure that the GBM cells were responsive to psychological concentrations of ACh, 1 µM ACh was used and a similar, but diminished response was observed (Figure S3). Alternatively, we compared the responses of D54 cells expressing the GCaMP3 calcium sensor [33] (a fusion protein including green fluorescent protein (GFP), calmodulin, and a peptide sequence from myosin light chain kinase) to those measured with Fluo-4AM and found similar responses (Figure S4). Based on these results, we concluded that GBM cells express functional mAChRs and nAChRs and cause an increase in [Ca^2+^]_i_ when activated.

### 3.3. GBM AChR Activation Has Minimal Effect on Proliferation or Migration

**Figure 3:**
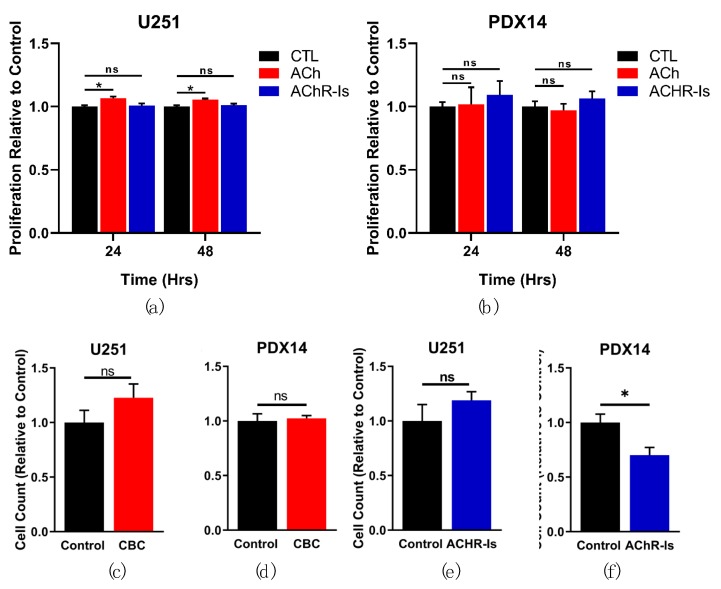
GBM AChR activation has minimal effect on proliferation and migration. The effect of AChR stimulation and inhibition on GBM proliferation and migration was evaluated with in vitro assays. Proliferation was measured in the U251 and PDX14 lines with an alamarBlue assay at 24 and 48 h in the presence of 1 mM ACh or AChRIs (1 µM atropine and 10 µM mecamylamine) (**a**, **b**). Migration of the U251 and PDX14 lines exposed to 100 µM CBC or AChRIs (1 µM atropine and 10 µM mecamylamine) was quantified in a transwell migration assay at 12 h (U251) and 48 h (PDX14) (**c**–**f**). A repeated measurement two-way ANOVA was used to analyze proliferation results: *** *p* = 0.0002 (**a**), *p* = 0.8969 (**b**). Dunnett’s multiple comparison test with a single pooled variance was used to analyze CTL vs. ACh/AChRIs in the proliferation assays (**a**, **b**): CTL vs. ACh @24 h *p = 0.0125, @48 h * *p* = 0.0357; CTL vs. AChRIs @24 h *p* = 0.8403, @48 h *p* = 0.7733 (**a**) CTL vs. ACh @24 h *p* = 0.9971, @48 h *p* = 0.9563; CTL vs. AChRIs @24 h *p* = 0.6656, @48 h *p* = 0.8206 (**b**). A two-tailed Student’s t test was used to analyze the Transwell migration results (**c**–**f**): *p* = 0.2377 (**c**), *p* = 0.7406 (**d**), *p* = 0.3067 (**e**), and *p = 0.0294 (**f**).

As Ca^2+^ is a key signaling molecule involved in the regulation of cell proliferation, we sought to determine if the increase in [Ca^2+^]_i_ with AChR stimulation would affect GBM cell proliferation. Proliferation in the presence of ACh was measured at 24 h and 48 h in the U251 and PDX14 cell lines with the alamarBlue proliferation assay (Thermo Fisher). A small significant increase was observed with the addition of ACh (6.59 ± 1.31% @24 h; 5.38 ± 1.10% @48 h) in the U251 line (Figure 3a). No significant change in proliferation was measured in the PDX14 line with ACh (Figure 3b). Alternatively, we measured proliferation in the presence of AChRIs and found no significant change in the proliferation of the U251 or the PDX14 lines (Figure 3a,b).

Increases in [Ca^2+^]_i_ have also been implicated in the migration of mammalian cells [34]. We wanted to determine if the observed increase in [Ca^2+^]_i_ upon AChR stimulation would lead to changes in GBM migration. We utilized a transwell migration assay (TMA), as previously described [35], to assess whether AChR activation leads to changes in GBM cell migration. Under conditions of AChR stimulation (hydrolysis-resistant ACh analog carbamylcholine: CBC) and AChR inhibition, no significant changes were seen in U251 (Figure 3c,d). In the PDX14 line, no significant changes occurred with AChR stimulation, but a significant decrease was observed when AChRs were inhibited (Figure 3e,f).

### 3.4. AChR Activation Significantly Enhances Glioblastoma Invasion

**Figure 4:**
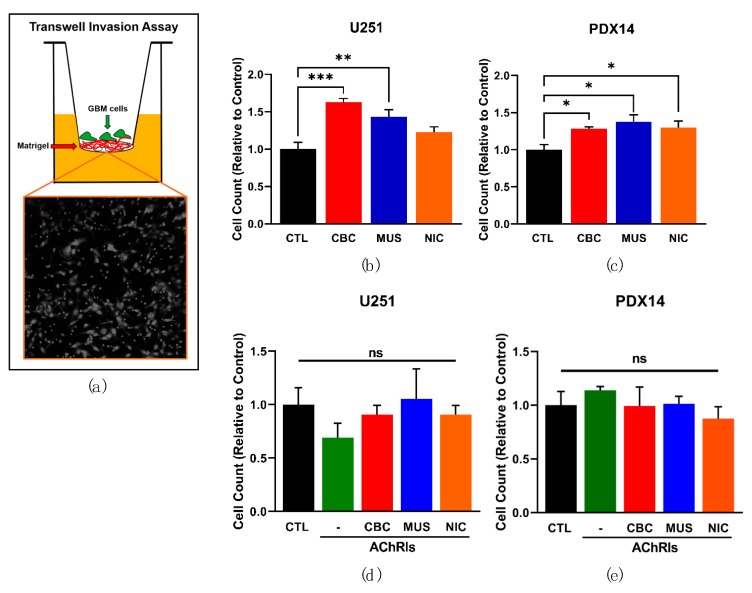
AChR activation significantly increases GBM cell invasion. A transwell invasion assay (TIA) was used to evaluate the invasiveness of GBM cells with AChR stimulation (100 μM CBC, 10 μM muscarine (MUS), 100 μM nicotine (NIC)) (**a**). TIA with U251 and PDX14 lines after 48 h (**b**, **c**). TIA with the U251 line in the presence of AChRIs (1 μM atropine and 10 μM mecamylamine) and AChR agonists (**d**, **e**). A one-way ANOVA was performed on the U251 TIA data *** *p* = 0.0008 (**b**), *p* = 0.6222 (**d**) and the PDX14 TIA data * *p* = 0.0191 (**c**), *p* = 0.6654 (**d**). Subsequently, Dunnett’s multiple comparison test with a single pooled variance was used to analyze CTL vs. CBC *** *p* = 0.0003, CTL vs. MUS ** *p* = 0.0066, CTL vs. NIC *p* = 0.1623 (**b**); CTL vs. CBC * *p* = 0.0488, CTL vs. MUS * *p* = 0.0101, CTL vs. NIC * *p* = 0.0391 (**c**).

After determining that AChRs have limited effects on GBM proliferation or migration, we hypothesized that AChRs could instead modulate invasion through the extracellular matrix (ECM) rich environment present in the brain. To answer this question, we utilized a transwell invasion assay (TIA) that mimics the ECM environment that tumor cells encounter in vivo. Transwell filters were coated with Matrigel, a mixture containing the ECM proteins typically found in the brain, and U251 and PDX14 cells were seeded upon this coating in unenriched medium after serum/growth factor starvation (Figure 4a). The cells at the top of the filter were exposed to AChR agonists at the time of plating to determine if AChR activation led to a difference in the invasive potential of the GBM cells. A significant increase in GBM cell invasion was observed with AChR stimulation, including selective stimulation of the mAChR and nAChRs for all conditions in the U251s and PDX14s, with the exception of nicotine in the U251s (Figure 4b,c). Next, we antagonized the AChRs with AChRIs in the presence of AChR agonists and found that, as expected, this abolished the ACh-mediated increase in both the U251 and PDX14 lines (Figure 4d,e).

### 3.5. GBM AChR Activation Increases Matrix Metalloproteinase-9 Activity

Based on these results, we hypothesized that the enhanced invasive capability of GBM cells with AChR stimulation could be due to an increase in matrix metalloproteinase (MMP) activity. The MMP family is collectively able to degrade all components of the ECM, and was thus a likely candidate to mediate enhanced invasion with AChR activation. MMPs are proteolytic enzymes that are largely responsible for all ECM remodeling that occurs within the body [36]. MMPs play critical roles during development and wound healing. However, various pathological conditions such as arthritis, stroke, and multiple sclerosis are characterized by the elevated expression of MMPs. Cancers, including GBMs, also have heightened expression of MMPs, allowing for the expansion of the tumor into and out of the primary organ [37]. In GBM, MMPs are highly expressed and this expression is inversely correlated with patient survival [37,38,39]. However, the gelatinases MMP-2 and MMP-9 are the most implicated MMPs [38,40,41].

**Figure 5:**
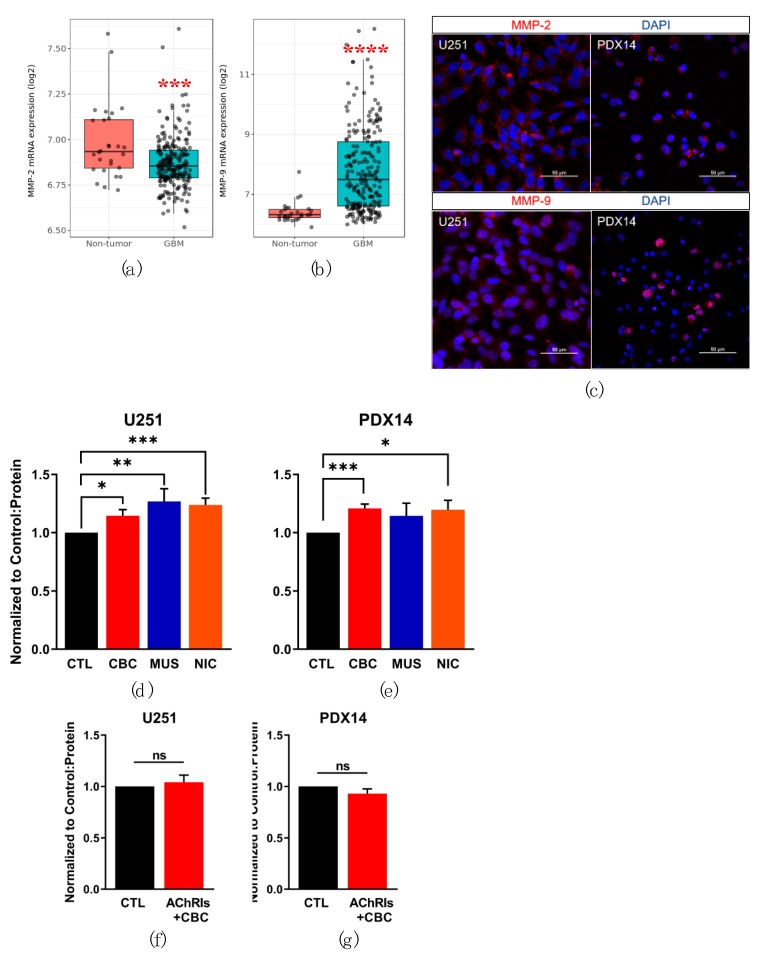
GBM AChR activation increases MMP-9 activity. REMBRANDT mRNA expression of *MMP-2* (**a**) and *MMP-9* (**b**) in non-tumor and GBM samples. Immunocytochemistry was used to confirm the expression of MMP-2 and MMP-9 in the U251 and PDX14 GBM lines (**c**). Gelatin zymography was used to measure MMP activity normalized to protein content and the control for each sample. Mature MMP-9 (67 kDa) activity after AChR stimulation in the U251 (**d**) and PDX14 (**e**) cell lines at 24 h, by AChR agonists (100 µM CBC, 10 µM muscarine (MUS), 100 µM nicotine (NIC)) MMP-9 activity with AChRIs (1 µM atropine and 10 µM mecamylamine) and 100 µM CBC in the U251 (**f**) and PDX14 (**g**) cell lines at 24 h. A pairwise t test with Bonferroni correction was used in (**a**) *p* = 0.00026 *** and (**b**) <0.0001 ****. A two-tailed Student’s t test was used to compare MMP-9 activity: CTL vs. CBC * *p* = 0.0159, CTL vs. MUS ** *p* = 0.0089, CTL vs. NIC *** *p* = 0.0003 (**b**); CTL vs. CBC *** *p* = 0.0002, CTL vs. MUS *p* = 0.2223, CTL vs. NIC *p = 0.0409 (**e**); CTL vs. AChRIs + CBC p = 0.5740 (**f**); CTL vs. AChRIs + CBC *p* = 0.2027 (**g**).

Furthermore, MMPs are synthesized as pro-enzymes and then packaged into vesicles and released from the cell, presumably by Ca^2+^-dependent exocytosis [42]. However, the mechanism of MMP release is poorly characterized in the literature. We hypothesized that AChR-mediated increases in [Ca^2+^]_i_ lead to elevated release and subsequent MMP activity. First, we used the Repository of Molecular Brain Neoplasia Data (REMBRANDT) dataset to compare mRNA expression of MMP-2 and MMP-9 in non-tumor control and GBM samples (Figure 5a,b). *MMP-2* mRNA expression was found to be significantly downregulated in the GBM samples (Figure 5a). In contrast, *MMP-9* expression was found to be significantly upregulated in the GBM samples (Figure 5b). We confirmed the expression of MMP-2 and MMP-9 with immunocytochemistry in both the adherent and xenograft lines (Figure 5c).

Next, we used gelatin zymography to evaluate the effect of AChR stimulation on MMP-2 and MMP-9 activity in the U251 and PDX14 cell lines. MMP activity was quantified following 24 h of AChR agonist exposure. In both lines, a significant increase in mature MMP-9 (67 kDa) activity occurred with AChR stimulation (Figure 5d,e). However, mature MMP-2 (45 kDa) activity was not detectable or no significant change in MMP-2 activity occurred with AChR stimulation (data not shown). Increases in MMP-9 activity occurred under conditions of AChR stimulation with universal AChR activator CBC, which was selected instead of ACh for its inability to be hydrolyzed during the prolonged incubation in these experiments. Muscarine and nicotine were also used for selective stimulation of mAChRs and nAChRs respectively. In the U251 cell line MMP-9 activity was found to be significantly upregulated with the addition of CBC, muscarine, and nicotine (Figure 5d). In the PDX14 line, MMP-9 activity was also significantly increased with the addition of CBC and nicotine, but not with the addition of muscarine (Figure 5e). As MMP-9 activity from GBM cells is considerable at the baseline, we wanted to determine if AChR inhibition would lead to a decrease in MMP activity and confirm that AChR activation was causal of the increase in MMP-9 activity. Thus, in addition to AChRIs, CBC was applied to the U251 and PDX14 cells and mature MMP-9 activity was measured. No significant difference between the control and AChRIs in combination with CBC samples were observed in either the U251 and PDX14 cell lines, suggesting that activation of AChRs is indeed required to increase MMP-9 activity (Figure 5f,g). Additionally, we applied AChRIs without AChR agonists and did not measure a significant difference between MMP-9 activity in the control and AChRIs conditions (Figure S5).

### 3.6. AChR-Induced MMP-9 Activity Is Dependent on Intracellular Ca^2+^

**Figure 6:**
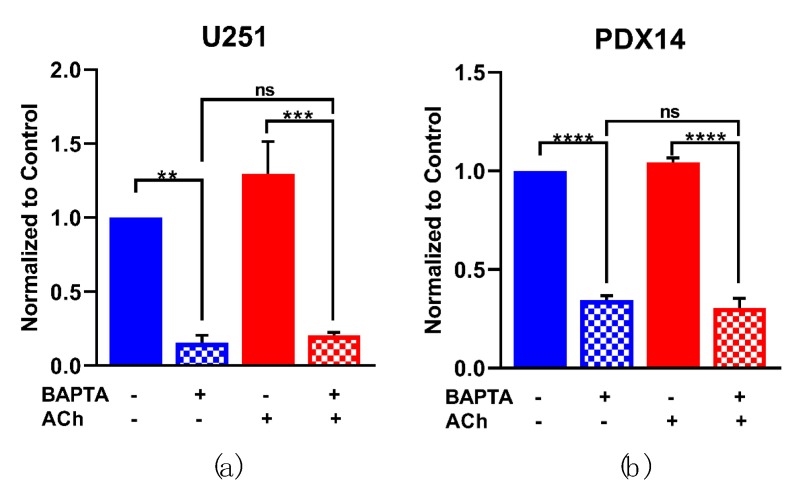
AChR-induced MMP-9 activity is dependent on intracellular Ca^2+^. [Ca^2+^]_i_ was chelated with cell permeant Ca^2+^-chelator BAPTA-AM (20 µM) in the U251 and PDX14 lines 30 min prior to the addition of 1 mM ACh. After 6 h, the medium was collected and gelatin zymography was performed (**a,b**). A one-way ANOVA was performed on the mature MMP-9 activity data from the U251s *** *p* = 0.0002 (**a**) and the PDX14s **** *p* < 0.0001 (**b**). Tukey’s multiple comparison test was used to compare the conditions: −BAPTA − ACh vs. +BAPTA − ACh ** *p* = 0.0032, −BAPTA + ACh vs. +BAPTA + ACh *** *p* = 0.0006, −BAPTA − ACh vs. +BAPTA + ACh *p* = 0.9886 (**a**); −BAPTA − ACh vs. +BAPTA − ACh **** *p* < 0.0001, −BAPTA + ACh vs. +BAPTA + ACh **** *p* < 0.0001, −BAPTA − ACh vs. +BAPTA + ACh *p* = 0.9002 (**b**).

To investigate whether the observed Ca^2+^ increases during AChR stimulation played a role in MMP-9 activity, we used the Ca^2+^ chelator BAPTA-AM to prevent increases in [Ca^2+^]_i_. Following a 30 min BAPTA-AM incubation, the U251 and PDX14 lines were stimulated with ACh and the conditioned-cell medium were collected after 6 h and MMP activity was quantified with gelatin zymography. With [Ca^2+^]_i_ chelated, mature MMP-9 activity was significantly reduced and pro- MMP-9 was not present in the U251 and PDX14 cells (Figure 6a,b). Upon addition to the Ca^2+^-chelated cells, ACh no longer significantly increased MMP-9 activity in either the U251 or PDX14 lines.

### 3.7. AChR-Induced MMP-9 Activity Increases GBM Cell Invasion

**Figure 7:**
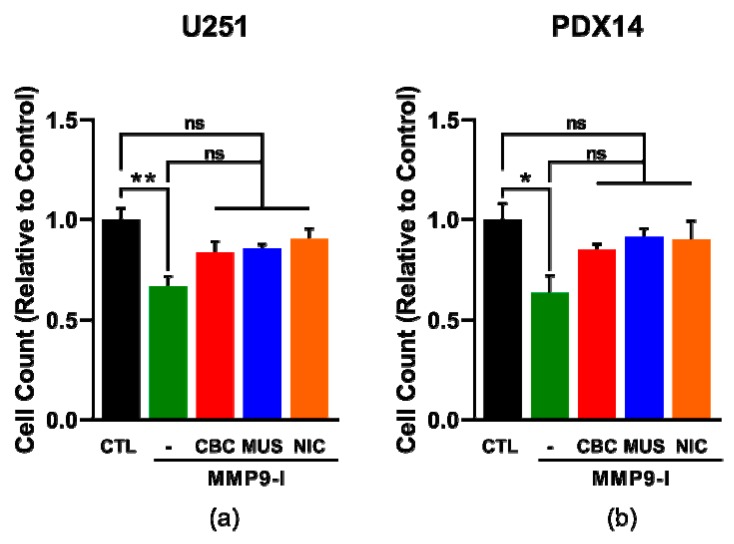
MMP-9 activity mediates the AChR-induced increase in GBM cell invasion. A transwell invasion assay (TIA) was used to evaluate the invasiveness of GBM cells with AChR stimulation and MMP-9 inhibition. TIA with the U251 (**a**) and PDX14 lines (**b**) after 48 h in the presence of MMP-9 Inhibitor I (MMP9-I, 100 nM). A one-way ANOVA was used for statistical analysis: ** *p* = 0.0061 (**a**); * *p* = 0.0296 (**b**). Subsequently, Tukey’s multiple comparisons test was used to analyze: CTL vs. CTL + MMP9-I ** *p* = 0.0028, CTL vs. CBC + MMP9-1 *p* = 0.1288, CTL vs. MUS + MMP9-I *p* = 0.2151, CTL vs. NIC + MMP9-I *p* = 0.6373, CTL + MMP9-I vs. CBC + MMP9-1 *p* = 0.1868, CTL + MMP9-I vs. MUS + MMP9-I *p* = 0.1151, CTL + MMP9-I vs. NIC + MMP9-I *p* = 0.0502 (**a**); CTL vs. CTL + MMP-1 * *p* = 0.0190, CTL vs. CBC + MMP9-1 *p* = 0.5856, CTL vs. MUS + MMP9-I *p* = 0.9059, CTL vs. NIC + MMP9-I *p* = 0.8482, CTL + MMP9-I vs. CBC + MMP9-1 *p* = 0.2659, CTL + MMP9-I vs. MUS + MMP9-I *p* = 0.0953, CTL + MMP9-I vs. NIC + MMP9-I *p* = 0.1226 (**b**).

Next, we wanted to determine if the enhanced invasion with AChR stimulation observed in the TIA was a result of the increased MMP-9 activity we also observed. To answer this question, we again used a TIA, but added a selective MMP-9 inhibitor (MMP-9 Inhibitor I, CAS 1177749-58-4) to confirm that MMP-9 was only responsible for the increased invasion observed with AChR stimulation. In the U251 and PDX14 lines, we found that MMP-9 inhibition led to a significant decrease in invasion without the addition of AChR agonists, but with the addition of AChR agonists, no significant difference was observed in comparison to the control (Figure 7a,b). These results confirm the role of MMP-9 in AChR-induced increases in GBM invasion.

### 3.8. AChRs Are Upregulated in Active Infiltration Zones in GBMs

**Figure 8:**
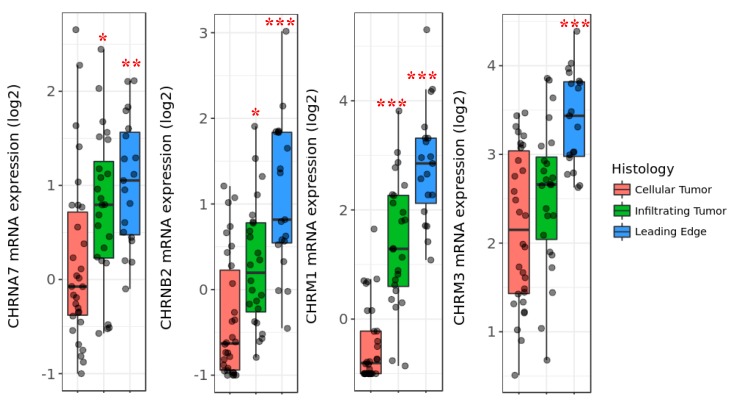
AChRs are upregulated in active zones of infiltration in GBM patients. mRNA expression of *CHRNA7*, *CHRNB2*, *CHRM1*, and *CHRM3* within cellular tumor (CT), infiltrating tumor (IT), and leading edge (LE) populations from GBM samples in the Ivy GAP dataset. Tukey’s honest significant difference test was used to compare the expression changes in the IT and LE populations in comparison to the expression within the CT population. *CHRNA7*: CT vs. LE *p* < 0.01 **, CT vs. IT *p* < 0.05 *; *CHRNB2*: CT vs. LE *p* < 0.001 ***, CT vs. IT *p* < 0.05*; *CHRM1*: CT vs. LE *p* < 0.001 ***, CT vs. IT *p* < 0.001 ***; *CHRM3*: CT vs. LE *p* < 0.001 ***, CT vs. IT *p* = 0.21.

Our data suggest that AChRs expressed on GBM cells stimulate the activity of MMP-9 and increase movement through complex ECMs. We thus hypothesized that there could be differential expression of these receptors in areas of active GBM invasion. Through the utilization of the Allen Institute Ivy Glioblastoma Atlas Project (Ivy GAP) database [24], we compared the expression of AChRs within the tumor mass (cellular tumor) to zones of active GBM infiltration (infiltrating tumor and leading edge). While the expression of many AChRs was not altered in comparison to the cellular tumor, we did find significant upregulations in the expression of *CHRNA7*, *CHRNB2*, *CHRM1*, and *CHRM3* in these areas (Figure 8). These findings suggest that these receptors may be upregulated in areas of active invasion to facilitate the enhanced activity of MMP-9 for the ECM modeling required during this process.

### 3.9. Are GBM Cells Capable of Synthesizing and Releasing ACh?

Our findings suggest that ACh is an important signaling molecule in GBM biology. Many cells besides cholinergic neurons are capable of synthesizing ACh, including epithelial cells in the lungs, endothelial cells, and various immune cells [43,44,45,46]. Malignant cells have also been shown to have the ability to make ACh [47,48]. We hypothesized that GBM cells might also be capable of synthesizing ACh, allowing the development of an autocrine loop in which ACh is made and released, thus stimulating further activity of MMP-9.

**Figure 9:**
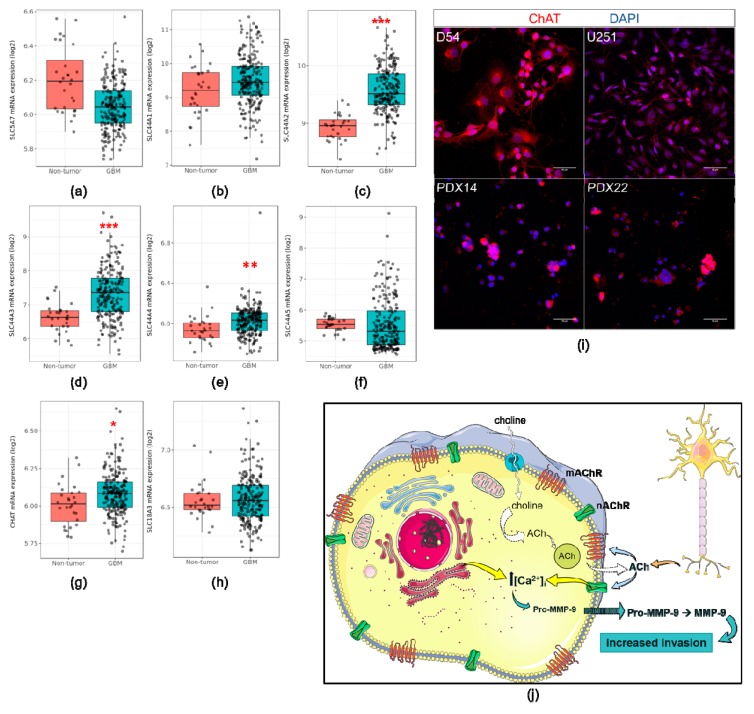
GBMs express other components of ACh signaling. mRNA expression of *SLC5A7* (**a**), *SLC44A1* (**b**), *SLC44A2* (**c**), *SLC44A3* (**d**), *SLC44A4* (**e**), *SLC44A5* (**f**), *CHAT* (**g**), and *SLC18A3* (**h**) in GBM samples from the REMBRANDT dataset. Immunocytochemical analysis of ChAT expression in the GBM cell lines (**i**). Proposed ACh autocrine signaling loop in GBM cells (**j**). Tukey’s honest significant difference test was used to compare the expression in the GBM samples in comparison to the non-tumor samples. *SLC44A2 p* < 0.001 *** (**c**), *SLC44A3 p* < 0.001 *** (**d**), *SLC44A4 p* < 0.01 ** (**e**), *CHAT p* < 0.05 * (**g**).

To determine whether this was the case, we evaluated the expression of various components of ACh synthesis and catabolism in another large GBM cohort: the Repository of Molecular Brain Neoplasia Data (REMBRANDT, https://gdoc.georgetown.edu/gdoc/) [23] (Figure 9). We elected to use this dataset instead of the TCGA dataset because the expression of our genes of interest could be evaluated on a single platform (microarray, Affymetrix U133 Plus 2.0) while maintaining a large sample size (GBM *n* = 219, non-tumor *n* = 28). First, we queried the expression of high- (*SLC5A7*) and low-affinity choline transporters (*SLC44A1-5*) in the GBM cohort. mRNA expression of all choline transporters was found within the GBM samples and in the case of *SLC44A2-4*, their expression was significantly upregulated (Figure 9a–f). The expression of choline acetyltransferase (*CHAT*), the enzyme responsible for ACh synthesis, was also found in the GBM samples and was significantly upregulated (Figure 9g). Expression of vesicular acetylcholine transporter (*SLC18A3*) was also confirmed in the REMBRANDT cohort (Figure 9h). At the protein level, we were able to confirm the expression of ChAT within the adherent and PDX lines with immunocytochemistry (Figure 9i). The collective expression of these enzymes, at the mRNA and protein level, suggests that GBMs are capable of synthesizing ACh, however, a quantitative assessment of release is still needed to definitively confirm this (Figure 9j).

### 3.10. Elevated CHRM3 Expression Shortens Survival Times in GBM Patients

Finally, we wanted to determine if AChR expression in GBMs could impact patient survival rates. Using the TCGA dataset, we found that heightened expression of *CHRM3* correlated to a significant decrease in patient survival times (*CHRM3* high 12.2 months vs. *CHRM3* low 15.4 months) (Figure S6). These findings provide further evidence that AChRs play an important role in GBM biology in situ.

## 4. Discussion

Neurotransmitters have known roles in the maintenance and regulation of the NSC population in the adult brain [8,49,50]. This population of cells has also been implicated as the likely cellular origin of GBMs, the most deadly of brain tumors [4,5,6,51]. However, the role that neurotransmitters could have on the biological behavior of GBMs is poorly understood. In this study, we sought to determine if AChRs influence GBM biology, as these tumors grow in areas densely innervated with cholinergic projections [11].

Using RNA-Seq expression analysis, we found that both the mAChR and nAChR subtypes were expressed in a large GBM cohort (TCGA). Most AChRs were, unsurprisingly, expressed at lower levels than the non-tumor control samples, as these samples are from unaffected brain areas where highly-expressing AChR populations of neurons and glial cells are present [52,53]. *CHRNA5*, *CHRNA10*, *CHRNB4*, *CHRND*, *CHRNE*, and *CHRNG*, however, were not expressed at levels significantly different from the non-tumor control samples. Furthermore, the expression of several AChRs was significantly elevated in the GBM samples including: *CHRNA1*, *CHRNA9*, and *CHRNB1*. The increased expression of *CHRNA1* and *CHRNB1* was surprising as these nicotinic subunits are generally considered part of “muscle” nicotinic receptors and their expression, to our knowledge, has not been characterized in the brain [54]. Whether this expression translates to a functional gain of protein expression, however, will only be possible with the development of selective antibodies, agonists, or antagonists. Characterization of the expression of AChRs in the NSCs population in adulthood has had limited investigation [9,55,56]. While these studies have been narrow in scope, the expression of several nicotinic and muscarinic receptors was confirmed in neural progenitors. Future studies with single cell RNA-Seq and protein analysis of human or murine NSC populations, however, would provide a definitive answer as to whether a parallel can been drawn between the expression profiles of AChRs in NSCs and GBM cells. At present, our data suggest that AChRs are expressed on GBM cells, possibly due to a gain of expression with malignant transformation or via the continued expression of AChRs on GBM-initiating cells.

We were unable to confirm protein expression of the AChRs via western blot due to the lack of commercially-available specific antibodies for the mAChR subtypes and nAChR subunits. Instead, we used time-lapse Ca^2+^ imaging as a readout of AChR functionality. Members of both the muscarinic and nicotinic families can cause increases in [Ca^2+^]_i_ upon activation [30,32]. However, activation of the M_2_ and M_4_ subtypes, which initiate G_i_ signaling, is not known to lead to changes in [Ca^2+^]_i_. For this reason, we cannot conclude definitively that these subtypes of mAChRs are functional via this method. Furthermore, we are limited to observing a collective response of the muscarinic (M_1_, M_3_, M_5_) and nicotinic subtypes due to the unavailability of subtype specific drugs. However, we did confirm the functionality of the homomeric α7nAChR on the D54 and U251 cell lines with the specific agonist PHA 543,613 hydrochloride (Tocris) (data not shown). With this method, we observed an increase in [Ca^2+^]_i_ upon activation of the AChRs collectively and with selective activation using nicotine and muscarine. These results support the previous findings of our group and those of others, where the expression of several AChRs in GBM cell lines were characterized [20,21,57,58]. However, these previous studies were limited, characterizing the expression of mAChRs only or the [Ca^2+^]_i_ response of ACh in a single GBM cell line. Our time-lapse studies provide an extended examination of AChRs in both GBM cell lines and PDX xenograft lines that recapitulate the heterogeneity of GBMs in situ. The calcium dynamics that we observed with the application of ACh varied between the cell lines (U251/D54) and the PDX xenograft lines (PDX14/PDX22), where a uniform response was observed in the cell lines and the PDX lines had a more diverse response across individual cells. We believe this difference is due in part to the heterogeneity of the cellular profile that PDX xenograft lines retain in culture, compared to the homologous nature of the cell lines. Calcium oscillations were observed under all conditions of AChR stimulation with varying frequency, most likely as a result of IP_3_-dependent Ca^2+^ release from internal stores [59]. Collectively, these results confirm the presence of functional AChRs including nicotinic and muscarinic subtypes on GBM cells.

Increases in [Ca^2+^]_i_ have been implicated in various cellular changes important in cancer progression including migration, proliferation, and invasion [60,61,62]. Application of ACh only produced a small (~5–6%) but significant increase in U251 proliferation. While significant, this small proliferation change is likely not biologically relevant, as proliferation from other molecules within the brain microenvironment have been found to increase proliferation at much higher rates [63,64,65]. Furthermore, antagonism of the AChRs did not produce any significant changes in proliferation within the U251 or PDX14 lines, suggesting that the AChRs that are expressed on GBM cells do not enhance or limit proliferation. There has been evidence to suggest that activation of the M_2_ muscarinic receptor inhibits cell cycle progression and thus proliferation in GBM cells [21,57]. We did not observe an inhibition of cell proliferation, however, we did not use selective mAChR agonists in our experiments. Our results suggest that unselective activation of the various mAChR and nAChR subtypes may cause conflicting signaling programs within GBM cells, such that no significant effect was observable. For example, G_i_ signaling activates “survival” mechanisms, rather than the “proliferate” programming of G_q_ signaling [66]. Additionally, the increases in [Ca^2+^]_i_ with AChR activation in GBM cells may not be sustained for long enough to mediate changes in cellular growth.

The hydrodynamic model of migration in glioma cells posits that cytoplasmic water can be fluxed, via ion channels, in and out of the cell to accommodate navigation into tight spaces within the brain parenchyma [67,68,69]. Several Ca^2+^-dependent ion channels have been implicated in this model including potassium channel KCa3.1 and chloride channel ClC-3 [70]. Moreover, other molecules present in the brain environment such as bradykinin, have been found to lead to the activation of these ion channels and increase GBM cell motility [58,71]. Surprisingly, we found no significant changes occurred with AChR stimulation and only in the PDX14 line was a significant decrease in migration found with AChR antagonism. These results suggest that AChRs do not initiate Ca^2+^-dependent ion channel-mediated changes in migration. Alternatively, there is evidence to suggest that mAChR activation inhibits migration [20]. While this stimulation led to an activation of Ca^2+^-dependent potassium channel KCa1.1, the link between the observed decrease in GBM cell migration and activation of this channel was not clear. Thus, we believe that the lack of an effect on GBM cell migration in the presence of ACh is likely to again be the result of contradictory intracellular signals and/or due to the relatively short-lived increase of [Ca^2+^]_i_ with ACh, as bradykinin-mediated GBM migration changes require prolonged Ca^2+^ oscillations [58]. On the other hand, this absence of a significant effect on GBM cell migration could be a consequence of membrane localization of the AChR receptors in relation to Ca^2+^-dependent ion channels. Bradykinin, a potent activator of GBM cell migration, was found to be expressed in close proximity to Ca^2+^-dependent ion channels at the leading edge of migratory cells [70]. However, whether AChRs are in close proximity to Ca^2+^-dependent ion channels that could mediate volumetric changes needed for migration is unknown. While the results of the migration studies suggest that they are not expressed together at the membrane, future studies are still needed to characterize the cellular location of AChRs in GBM cells.

GBM invasion requires enzymatic reorganization of the ECM to allow movement through complex environments such as the brain parenchyma or the perivascular space. We found that GBM cells invaded more significantly in the presence of AChR agonists. The observed differences in the effect of AChR activation on migration versus invasion suggests that there is an enhanced capacity to navigate through complex matrices, which requires the involvement of ECM remodeling enzymes. In light of these results, we considered the possibility that AChR-mediated Ca^2+^ increases lead to an increase in MMP activity. While both MMP-2 and MMP-9 were found to be highly expressed in the adherent and xenograft lines, only *MMP-9* expression was found to be significantly upregulated in the REMBRANDT dataset. Moreover, AChR activation led to a significant increase in only MMP-9 activity. MMP-9 expression is known to be upregulated in response to various stimuli including epidermal growth factor (EGF), interleukin-8 (IL-8), and tumor necrosis factor alpha (TNF-α) [72,73,74].

Our findings are supported by a limited number of studies that have found ACh stimulation increases MMP activity and expression in a mouse neuroblastoma cell line and human colon cancer cell line, respectively [75,76]. However, this study is the first to link the activation of AChRs to increased GBM invasion and MMP-9 activity. An important question to be addressed in the future, however, is whether AChR activation leads to changes in the transcriptional or translational expression of MMP-9, as other modulators such as astrocyte elevated gene-1 (AEG-1) have been found to elicit [77]. Our Ca^2+^ chelation experiments provide evidence that the acute increase in [Ca^2+^]_i_ is essential for MMP-9 activity in both the basal and stimulated conditions, however, future experiments that modulate the transcription and translation of MMP-9 would allow for a clear understanding of the potential influence of AChR activation in these processes.

Chelation of [Ca^2+^]_i_ demonstrated that the increase in MMP-9 activity occurs via a Ca^2+^-dependent mechanism, regardless of the activation status of the AChRs. These results may also suggest that intracellular calcium is required for MMP-9 release. As previously mentioned, MMP release is presumed to occur via calcium-dependent exocytosis, however, evidence in support of this mechanism of release is still lacking. While our results do not provide a definitive confirmation, the significant decrease in the presence of both pro- and mature MMP-9 in the external solution would suggest that the amount of MMP-9 outside the cell is greatly diminished. Thus, these results posit that the release of MMP-9 is being inhibited by the chelation of [Ca^2+^]_i_. Future studies with verification by proteolytic analysis could prove critical to address these outstanding questions and whether this finding can be extrapolated to other MMPs.

AChR stimulation caused a significant increase in GBM cell invasion. As noted previously, this was in stark contrast to our transwell migration results, where no significant increase in migration was observed. We believe that this is due to an absence of ion channel activation, but instead, the enhanced activity of MMP-9, as a specific MMP-9 inhibitor (CAS 1177749-58-4) mitigated this effect. These results affirm the highly interactive relationship between GBM cells and the microenvironment they inhabit. While other examples of the influence of the microenvironment on GBM behavior have been increasingly documented, these results provide novel insights into the role of a neurotransmitter. Moreover, this study and another recent study [14] have identified neurotransmitters as key modulators of GBM progression.

In light of our discovery of the role that AChRs play in GBM invasion, we questioned whether these in vitro findings would translate to differential expression of these receptors in areas of active infiltration in situ. Utilizing the Ivy GAP dataset, we found a significant upregulation of several AChRs. This upregulation suggests that these receptors play a critical role in the promotion of GBM progression, and potentially via the mechanism we have proposed in this study. However, experimental confirmation is still needed to affirm this correlation, and the question of what is leading to the upregulation of these receptors is yet to be answered. Remarkably, we also found that elevated expression of the muscarinic M_3_ receptor (*CHRM3*) correlated with decreased patient survival. Our studies would suggest that this is a result of the enhanced ability of these tumors to invade in comparison to tumors that have lower expression. Again, experimentation is needed to validate this correlation. Clinically, a selective muscarinic M_3_ receptor antagonist has been FDA approved to treat overactive bladder, however, it appears that this drug does not cross the blood–brain barrier [78,79]. Should selective antagonists with bioavailabity in the brain be developed, these could prove to be advantageous therapeutics in GBM.

Autocrine signaling loops are often hijacked by malignant cells, resulting in tumor progression. For example, platelet-derived growth factor, nerve growth factor, and EGF have been implicated in autocrine loops in several malignancies [80,81,82,83]. Here, our investigation provides preliminary evidence that autocrine ACh promotes GBM malignancy (Figure 9j). We confirmed the mRNA expression of several of the enzymes required for ACh synthesis including choline transporters, choline acetyltransferase, and vesicular acetylcholine transporters. Surprisingly, we found a significant upregulation of *SLC44A2*-*A4* and *CHAT* expression in the GBM samples. Elevated choline metabolism is well documented in many cancers, where the transport of choline into the cell is considered the rate-limiting step and is considered a therapeutic target [84,85]. Choline is a major component of membrane lipids and is thus critical for cell proliferation and viability. While elevated choline transport in GBM could be solely utilized for membrane synthesis, our findings that ChAT is expressed and significantly upregulated in GBMs suggest that choline transport could have a multifaceted purpose in GBM cells. While analytical measurements of conditioned-media from GBM cells are still needed to conclude that ACh synthesis is definitively occurring, this is not the first example of GBM cells producing neurotransmitters [14,15]. Again, an interesting question to answer would be whether the expression of these enzymes is an example of a gain-of-function or simply the retention of expression that was already present in the GBM cell-of-origin. There is some indication in the literature that this could be the latter, where the synthesis of inhibitory neurotransmitter GABA has been documented, however, more investigation is required [86].

## 5. Conclusions

In conclusion, this study is the first comprehensive characterization of AChRs in a large GBM sample cohort, GBM cell lines, and PDX xenograft lines. Our findings suggest that functional AChRs are expressed on GBM cells and play a role in promoting the invasiveness of these tumors via the increased activity of MMP-9. Additionally, we demonstrated that MMP-9 activity is dependent on intracellular calcium. Expression of several AChRs was found to be significantly upregulated at active zones of GBM infiltration in situ and elevated expression of the muscarinic M_3_ receptor was found to significantly correlate with decreased patient survival. Finally, we found that GBMs express other enzymes required to make ACh, suggesting that these tumors could be capable of synthesizing ACh and thus through autocrine signaling, increase invasiveness into the ECM rich brain environment. Collectively, these findings present a novel signaling axis within GBMs that could be targeted therapeutically to mitigate one of the predominant challenges limiting the effective treatment of these deadly tumors.

## Figures and Tables

**Table 1:** AChR expression in TCGA dataset. mRNA expression of GBM patient samples with RNA-Seq values expressed as fold changes (log2) via the GlioVis platform. A significant upregulation was observed in the GBM samples in comparison to the non-tumor control samples with a pairwise t test with Bonferroni correction for *CHRNA1* (* *p* = 0.024), *CHRNA9* (** *p* = 0.0092), and *CHRNB1* (*** *p* = 0.00014).

Gene Name	GBM Samples (*n* = 156) mRNA Expression (mean ± SD)	Non-Tumor Samples (*n* = 4) mRNA Expression (mean ± SD)
*CHRM1*	5.00 ± 2.35	11.52 ± 0.47
*CHRM2*	0.43 ± 1.49	5.75 ± 0.70
*CHRM3*	4.79 ± 1.86	8.67 ± 0.41
*CHRM4*	3.74 ± 1.27	7.39 ± 0.39
*CHRM5*	1.93 ± 1.28	4.64 ± 1.31
*CHRNA1*	5.65 ± 2.58 *	2.71 ± 0.34
*CHRNA2*	1.64 ± 1.90	7.61 ± 0.57
*CHRNA3*	3.14 ± 1.88	5.02 ± 0.15
*CHRNA4*	5.06 ± 2.37	9.96 ± 0.19
*CHRNA5*	5.41 ± 1.11	4.35 ± 0.61
*CHRNA6*	2.15 ± 1.61	3.93 ± 0.81
*CHRNA7*	3.09 ± 1.72	5.25 ± 1.00
*CHRNA9*	5.76 ± 2.11 **	2.96 ± 1.29
*CHRNA10*	3.76 ± 0.81	3.32 ± 0.60
*CHRNB1*	8.47 ± 0.56 ***	7.31 ± 0.31
*CHRNB2*	7.22 ± 1.54	11.23 ± 0.20
*CHRNB3*	−0.39 ± 0.98	3.25 ± 0.79
*CHRNB4*	1.43 ± 1.71	2.93 ± 0.60
*CHRND*	0.28 ± 1.29	−0.77 ± 0.46
*CHRNE*	4.21 ± 1.05	3.63 ± 0.67
*CHRNG*	−0.61 ± 0.64	−0.09 ± 0.75

## Supplementary Materials

The following are available online at https://www.mdpi.com/2073-4409/8/10/1203/s1, Table S1: Annotated mutations of AChR genes in the TCGA GBM dataset; Figure S1: Expression of AChRs in the GBM cell and xenograft lines; Figure S2: Calcium responses to AChR stimulation in D54 and PDX22 lines; Figure S3: Calcium response to 1 μM Ach; Figure S4: Calcium response to AChR stimulation in the D54-GCaMP cell line; Figure S5: MMP-9 release is not affected by AChR inhibition; Figure S6: Increased *CHRM3* expression correlates to decreased survival times.
