# Supplementary material for: Acetylcholine Receptor Activation as a Modulator of Glioblastoma Invasion
Source: Cells. 2019 Oct 5;8(10):1203. doi: 10.3390/cells8101203 (PMC6829263; doi:10.3390/cells8101203)
Supplement: Supplementary file 1 [file cells-08-01203-s001.pdf]

cells

Supplemental Table and Figures for:

Acetylcholine Receptor Activation as a Modulator of Glioblastoma Invasion

Emily G. Thompson [1,2] and Harald Sontheimer [1,3,*]

[1] Glial Biology in Health, Disease and Cancer Center, Fralin Biomedical Institute at Virginia Tech-Carilion, Roanoke, VA 24016
[2] Department of Neurobiology, University of Alabama at Birmingham, Birmingham, AL 35294
[3] School of Neuroscience, Virginia Polytechnic Institute and State University, Blacksburg, VA 24061
* Correspondence: sontheim@vt.edu

Table S1. Annotated mutations of AChRs in TCGA GBM dataset.

Sample	Gene	Mutation Type	Amino Acid change	Chromosome
TCGA.76.6660	CHRM1	Missense	G450D	11
TCGA.02.2485	CHRM2	Missense	K376E	7
TCGA.28.5207	CHRM2	Missense	G238R	7
TCGA.76.6285	CHRM2	Missense	R363H	7
TCGA.06.0155	CHRM3	Missense	A495V	1
TCGA.06.5858	CHRM4	Missense	R473Q	11
TCGA.32.4210	CHRM4	Missense	A263T	11
TCGA.06.0649	CHRNA10	Missense	R387Q	11
TCGA.19.2629	CHRNA10	Missense	M303V	11
TCGA.06.0744	CHRNA4	Missense	G625D	20
TCGA.76.4931	CHRNB2	Missense	Q59*	1
TCGA.06.0645	CHRNB4	Missense	D259N	15
TCGA.19.5953	CHRNB4	Missense	V448I	15
TCGA.06.2570	CHRND	Missense	T84K	2
TCGA.19.2631	CHRND	Missense	Y239H	2
TCGA.16.1048	CHRND	Missense	R244H	2
TCGA.76.6657	CHRNG	Missense	A44T	2

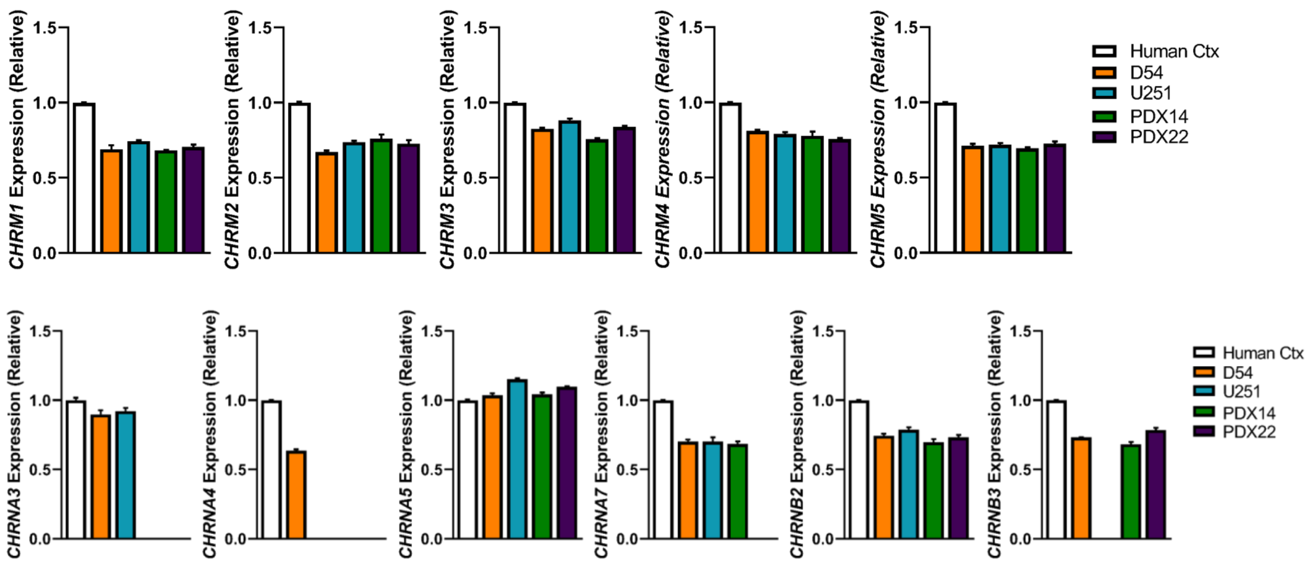

Figure S1. Expression of AChRs in GBM cell and xenograft lines. qPCR analysis of AChR expression (*CHRM1-5*; *CHRNA3-7*; *CHRNB2-4*) in adherent GBM cell lines (D54 and U251) and patient-derived xenograft (PDX) lines (PDX14 and PDX22). Gene expression is expressed relative to internal reference gene *IPO8* and then relative to control tissue from human brain cortex (Human Ctx). Expression was not found for *CHRNA3* in the PDX14 and PDX22 lines, *CHRNA4* in the U251, PDX14, and PDX22 lines, *CHRNA7* in the PDX22 line, *CHRNB3* in the U251 line, *CHRNB4* in all cell and PDX lines.

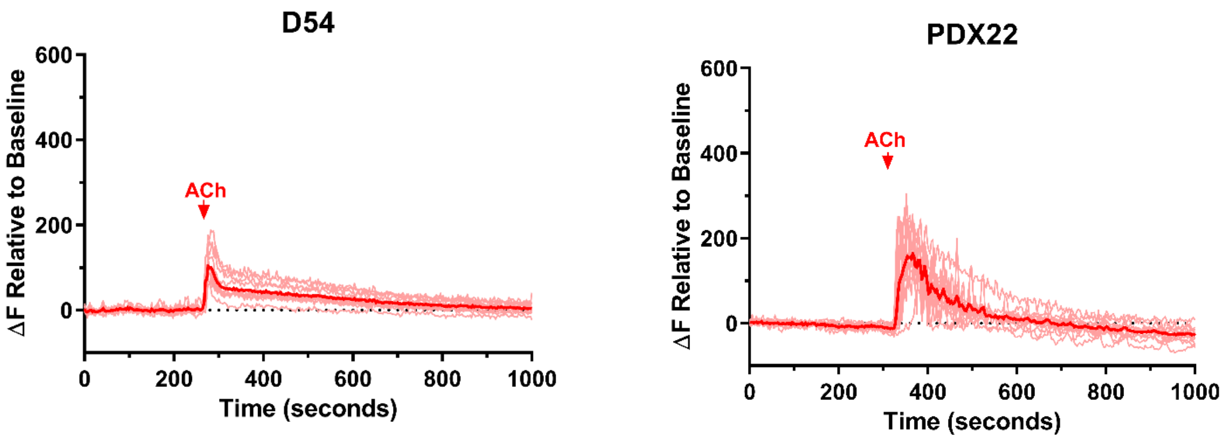

(a) (b)

Figure S2. Calcium responses to AChR stimulation in D54 and PDX22 lines. Representative graphs of $[Ca^{2+}]_i$ changes with 1mM ACh application in the D54 and PDX22 lines **(a, b)**.

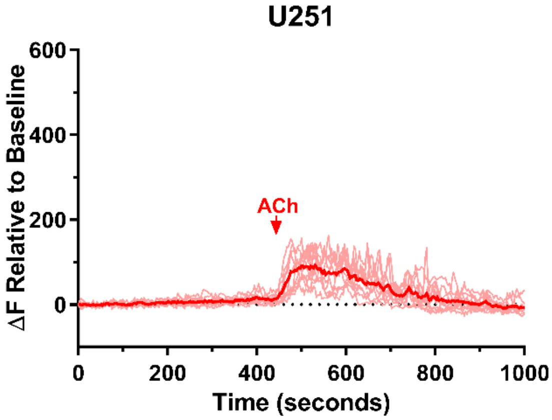

Figure S3. *Calcium response to 1μM ACh.* [Ca^{2+}]$_i$ changes in U251 line with 1μM ACh application.

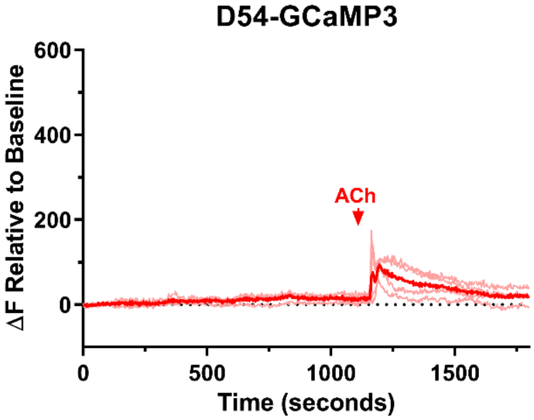

Figure S4. Calcium response to AChR stimulation in D54-GCaMP cell line. [Ca^{2+}]$_i$ changes with application of 1mM ACh in the D54 cells utilizing genetically encoded calcium sensor, GCaMP3.

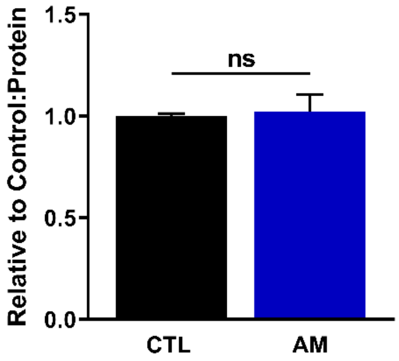

Figure S5. MMP-9 activity is not affected by AChR inhibition. AChR inhibitors (AM, 1μM atropine and 10μM mecamylamine) were applied to U251 cells and the conditioned media was collected at 24hr for gelatin zymography analysis. MMP-9 activity was not significantly difference between the control and AM condition. A student's test was used for statistical analysis, $p=0.8045$.

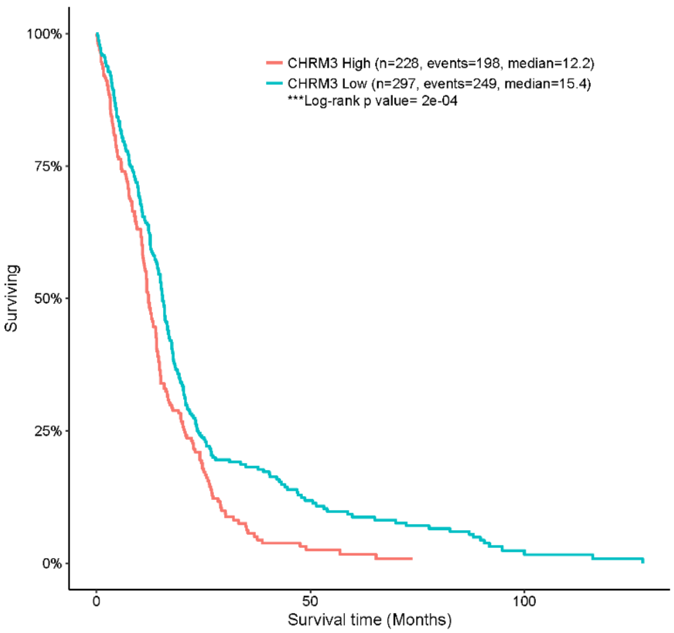

Figure S6. Increased CHRM3 expression correlates to decreased survival times. Kaplan-Meier survival curve of TCGA GBM patients grouped by *CHRM3* mRNA expression (HG-U133A), high vs. low expression was defined by calculating the optimal cutoff value of 5.76 with maximally selected rank statistics. The log-rank test was used to compare survival differences between GBM patients with high vs. low *CHRM3* expression, $p<0.001$***.
